# Combination of Immune Checkpoint Inhibitors and Radiotherapy for Advanced Non-Small-Cell Lung Cancer and Prostate Cancer: A Meta-Analysis

**DOI:** 10.1155/2021/6631643

**Published:** 2021-01-05

**Authors:** Zexi Xu, Jia Feng, Yiming Weng, Yao Jin, Min Peng

**Affiliations:** Department of Oncology Center, Renmin Hospital of Wuhan University, Wuhan 430060, China

## Abstract

**Objectives:**

Immune checkpoint inhibitors (ICI) combined with radiotherapy (RT) have emerged as a breakthrough therapy in the treatment of various cancers. The combination has a strong rationale, but data on their efficacy and safety are still limited. Hence, we comprehensively searched the database and performed this study to elucidate the clinical manifestations of this combined strategy.

**Methods:**

We performed a meta-analysis of randomized trials that compared ICI plus RT with placebo plus RT or ICI alone for the treatment of advanced nonsmall-cell lung cancer (NSCLC) and prostate cancer. The outcomes included overall survival (OS), progression-free survival (PFS), disease control rate (DCR), and treatment-related adverse events. A fixed-effects or random-effects model was adopted depending on between-study heterogeneity.

**Results:**

Three trials involving 1584 patients were included. ICI plus RT was significantly associated with improvement of OS (hazard ratio [HR] = 0.81, 95% confidence interval [CI] = 0.70–0.94, *P*=0.004), PFS (HR = 0.64; 95% CI 0.56–0.72, *P* < 0.00001), and DCR (relative risk [RR] = 1.38; 95% CI 1.03–1.84, *P*=0.03). A significant predictor for PFS with the combination of ICI and RT was age, as a significant improvement in PFS (HR = 0.49; 95% CI 0.37–0.64, *P* < 0.00001) was observed in NSCLC patients aged under 65 years. In safety analyses, patients receiving ICI plus RT had a significantly higher incidence of dyspnea (RR = 2.43; 95% CI 1.16–5.08, *P*=0.02) and pneumonitis of grade 3 or higher (RR = 2.78; 95% CI 1.32–5.85, *P*=0.007).

**Conclusion:**

The combination of ICI and RT was associated with improved OS, PFS, and DCR. Patients under 65 years will be the dominant beneficiaries. However, the incidence of dyspnea and pneumonia of grade 3 or higher also increased, which deserves our vigilance.

## 1. Introduction

Over recent decades, immune checkpoint inhibitors (ICI) are considered a major advance in the treatment of various advanced cancers. Targets of ICI therapy include cytotoxic T lymphocyte antigen-4 (CTLA-4), programmed cell death protein 1 (PD1), and programmed death-ligand 1 receptor (PD-L1). However, actually, the majority of patients treated with ICI do not achieve objective responses, and most neoplasm regressions are partial rather than complete [1]. With the recent success of radiotherapy (RT) combined with immunotherapy in animal models of various types of cancer, there has been renewed interest in the combination of ICI with RT [2–7]. However, it is not clear whether RT can enhance the effects of ICI in human patients, and the safety of combined therapy has not been fully confirmed. We performed a systematic review of the literature and reported the outcomes of patients with solid neoplasm treated with ICI and RT.

## 2. Materials and Methods

We conducted this meta-analysis on the basis of the PRISMA statement [8]. All analyses were conducted based on previously published studies; thus, no ethical approval and patient consent are required.

### 2.1. Search Strategy

Two reviewers independently completed the search. They were searched, with no time restrictions, the following databases for relevant English language literature: PubMed (MEDLINE), Embase, the Cochrane Central Register of Controlled Trials (CENTRAL), US-clinical trials, and China National Knowledge Infrastructure Database (CNKI). The following keywords were used: “Neoplasms,” “Neoplasia,” “Cancer,” “Immunotherapy,” “Programmed Cell Death 1 Receptor,” “Programmed Cell Death 1 Ligand 1,” “CTLA-4 Antigen,” “Durvalumab,” “Pembrolizumab,” “Atezolizumab,” “Nivolumab,” “Tremelimumab,” “Ipilimumab,” “Radiotherapy,” “randomized controlled trial.” The electronic database search will be supplemented by a manual search of the reference lists of included articles.

### 2.2. Inclusion Criteria

All relevant articles underwent evaluation for eligibility by two investigators independently. The relevant clinical trials were selected carefully based on the following criteria: (1) population: participants with histologically confirmed solid tumors; (2) intervention: PD-1/PD-L1/CTLA-4 Inhibitors combined with RT (or chemoradiotherapy), RT were applied prior to immunotherapy or during immunotherapy, and the types of RT include conventional radiotherapy and stereotactic radiotherapy; (3) comparison: ICI or RT alone; (4) outcomes: overall survival (OS), progression-free survival (PFS), clinical efficacy and treatment safety; (5) study design: randomized controlled trials (RCTs). The following exclusion criteria were considered: insufficient data were available to estimate the outcomes; the size of each arm was less than ten patients; animal studies and nonrandomized studies.

### 2.3. Data Extraction

Literature screening and data extraction were carried out by two independent reviewers. The discrepancy was resolved by discussion between the two of us. If the two authors could not reach a consensus, another author made the decision. For each study, the following details were extracted: the name of the first author, year of publication, cancer type, treatment arm and control arm, sample size, dose, the hazard ratio (HR), and 95% confidence interval (CI) for PFS (defined as the time from randomization to the first documented disease progression or death), and OS (defined as the time from randomization to death), disease control rate (DCR), number of patients who experienced adverse effects (AEs). We also extracted HR and 95% CI for PFS of the intention-to-treat population and the following predefined subgroups: age (<65 vs. ≥65 years), sex (female vs. male), tumor histology (squamous nonsmall-cell lung cancer [NSCLC] vs. nonsquamous NSCLC).

### 2.4. Quality Assessment and Statistical Analysis

Two investigators used the risk of bias tool (Cochrane Handbook for Systematic Reviews of Interventions, Version 5.1.0) to independently assess the quality of the trials. Sequence generation, allocation concealment, blinding, incomplete data, selective reporting, and other sources of bias were assessed. A trial with a high risk of bias for any one or more key domains was considered as “high risk.” A trial with a low risk of bias for all key domains was considered as “low risk.” Otherwise, it was considered as “unclear.” Disagreements between the two investigators were resolved by discussion with a third investigator.

### 2.5. Statistical Analysis

Statistical analysis was performed using the software Review Manager 5.3 (Cochrane Collaboration, Oxford, UK); HR and their 95% CI were used as summary statistics for OS and PFS in the present meta-analysis. For dichotomous outcomes, we extracted the number of events and participants in each treatment arm to estimate the relative risk (RR). *I*^2^ was used to assess heterogeneity across studies, with *I*^2^ values of 0%, 25%, 50%, and 75% representing no, low, moderate, and high heterogeneity, respectively. According to the Cochrane review guidelines, if severe heterogeneity was present at *I*^2^ > 50%, the random effect models were chosen. Otherwise the fixed effect models were used. The possibility of publication bias was estimated by funnel plots. A *P* value <0.05 was considered statistically significant.

## 3. Results

### 3.1. Search Results

**Figure 1:**
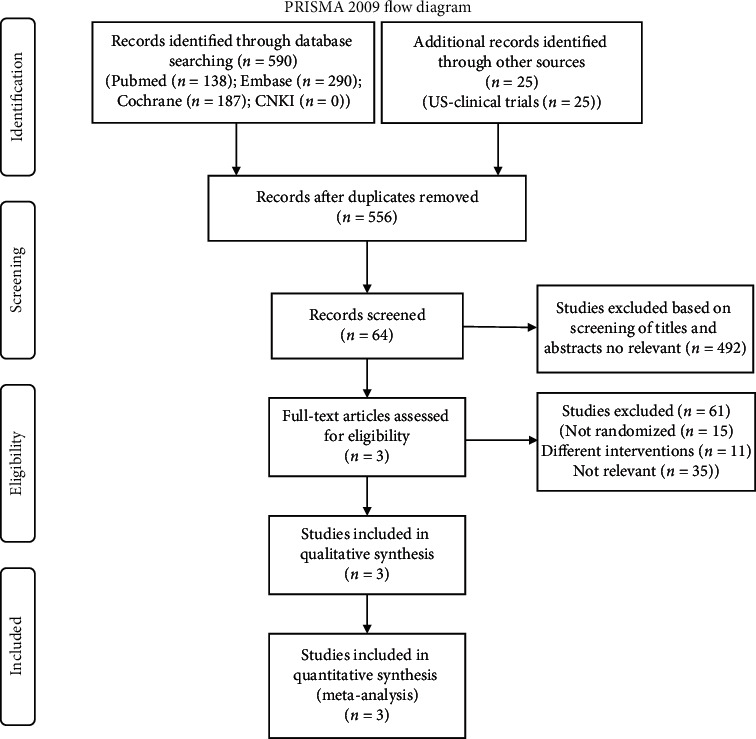
Flow diagram of included and excluded studies. After successively applying the inclusion and exclusion criteria, 3 RCTs were included, but no clinical trials with usable data.

Figure 1 illustrates the PRISMA flow chart for study inclusion and exclusion criteria. The database search yielded 615 records. After deleting the duplicate results, a total of 556 records remained for title and abstract review. Of these, 64 trials were selected for full-text examination. Three studies fulfilled the inclusion criteria and were suitable for the analysis.

### 3.2. Characteristics of Included Studies

In this meta-analysis, we included three RCTs involving 908 patients in the experimental groups and 676 patients in the control groups. One study evaluated chemoradiotherapy combined with durvalumab versus chemoradiotherapy with placebo in patients with NSCLC [9], one study compared RT plus ipilimumab versus RT with placebo in patients with prostate cancer [10], and one study assessed pembrolizumab after RT versus pembrolizumab alone in NSCLC [11]. The characteristics of the studies are reported in Table 1.

### 3.3. Methodological Bias of Included Studies

**Figure 2:**
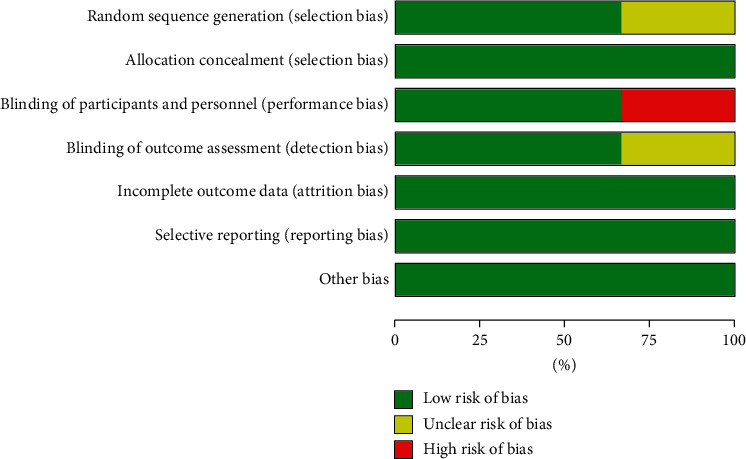
Risk of bias graph.

Among the three studies, two studies clearly reported the random allocation methods, and all three studies provided detailed information concerning allocation concealment, blinding of participants and personnel, and outcome assessment.  Figure 2 summarizes the methodological quality of the included trials.

### 3.4. Statistical Analysis of Efficacy Outcomes

#### 3.4.1. Overall Survival and Progression-Free Survival

**Figure 3:**
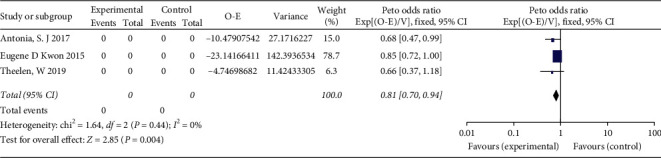
Forest plot for the meta-analysis of HR of OS for ICI+RT in solid tumors. HR: hazard ratio; ICI: Immune checkpoint inhibitors; OS: overall survival; RT: radiotherapy.

**Figure 4:**
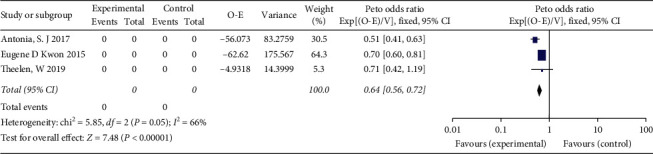
Forest plot for the meta-analysis of HR of PFS for ICI+RT in solid tumors. HR: hazard ratio; ICI: Immune checkpoint inhibitors; PFS: progression-free survival; RT: radiotherapy.

The combination of ICI with RT vs. placebo with RT or ICI alone is shown in Figures 3 and 4. The relative benefit in OS of receiving ICI and RT was significant (HR = 0.81, 95% CI 0.70–0.94, *P*=0.004). The analysis of PFS showed that the addition of immunotherapy to RT reduces the risk of recurrence and metastasis by 36% in patients with NSCLC and prostate cancer (HR = 0.64; 95% CI 0.56–0.72, *P* < 0.00001).

#### 3.4.2. Disease Control Rate

**Figure 5:**
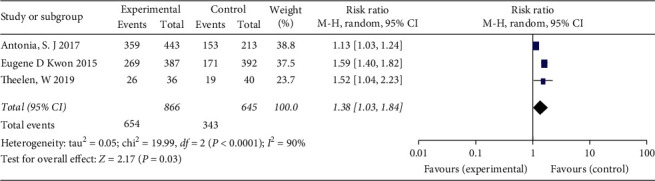
Forest plot for the meta-analysis of RR of DCR for ICI+RT in solid tumors. DCR: disease control rate; ICI: Immune checkpoint inhibitors; RR: relative risk; RT: radiotherapy.

RR for the DCR was available for three trials. The pooled analysis of DCR using the random-effects model is shown in Figure 5. There was statistical heterogeneity among the studies (*I*^2^ = 90%). The results showed that RT combined with ICI significantly increased the DCR (RR = 1.38; 95% CI 1.03–1.84, *P*=0.03).

#### 3.4.3. Subgroup Analysis

**Figure 6:**
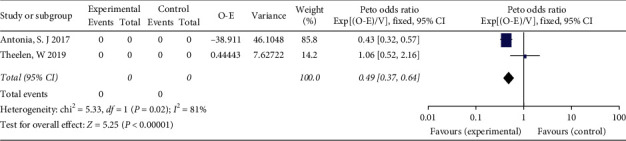
Forest plot for the subgroup analyses of HR of PFS for ICI+RT in NSCLC patients under 65. HR: hazard ratio; ICI: Immune checkpoint inhibitors; NSCLC: nonsmall-cell lung cancer; PFS: progression-free survival; RT: radiotherapy.

**Figure 7:**
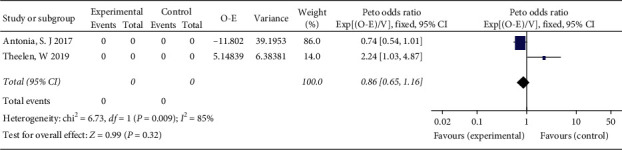
Forest plot for the subgroup analyses of HR of PFS for ICI+RT in NSCLC patients older than 65. HR: hazard ratio; ICI: Immune checkpoint inhibitors; NSCLC: nonsmall-cell lung cancer; PFS: progression-free survival; RT: radiotherapy.

We performed a subgroup analysis by grouping NSCLC patients based on whether they were older than 65 years. A significant improvement was observed in PFS (HR = 0.49; 95% CI 0.37–0.64, *P* < 0.00001) in patients aged under 65 years who received ICI combined with RT (Figure 6), but no significant difference in PFS was observed in patients older than 65 years (HR = 0.86; 95% CI 0.65–1.16, *P*=0.43) (Figure 7). There was no significant difference in PFS between genders or pathological types of NSCLC.

### 3.5. Statistical Analysis of Safety Outcomes

#### 3.5.1. Any Grade Adverse Events and Grade 3–5 Adverse Events

**Figure 8:**
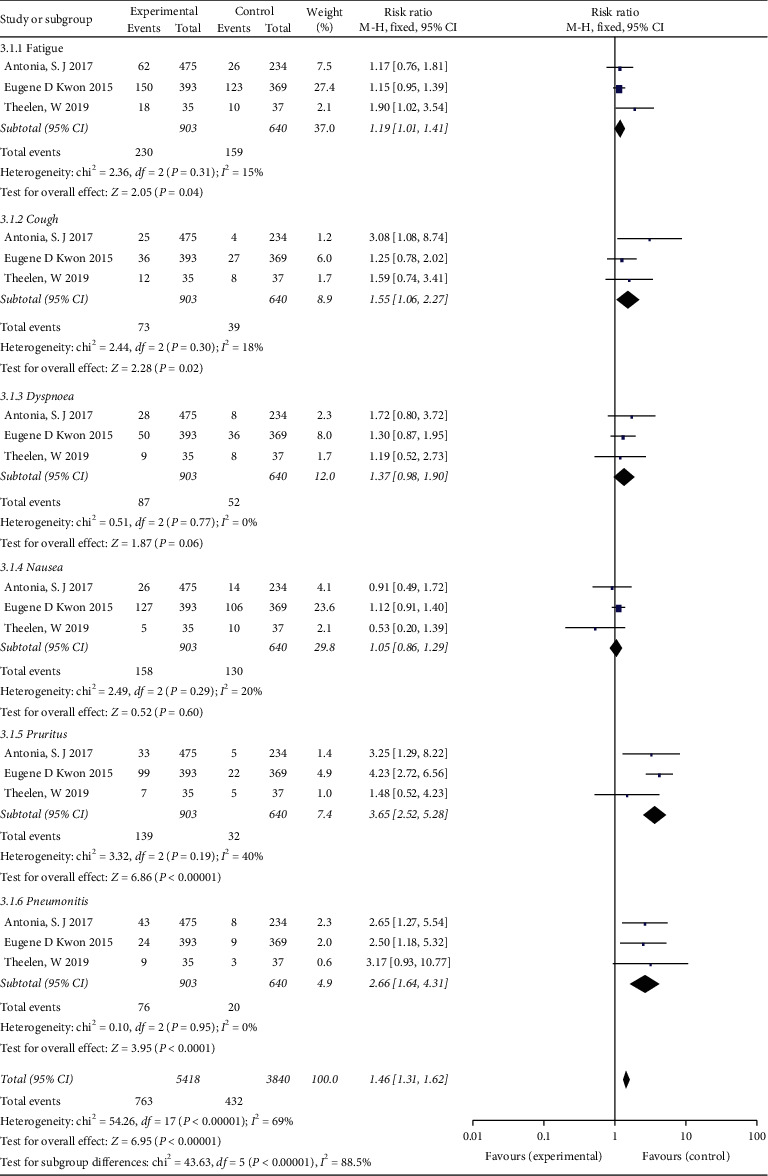
Forest plot for the subgroup analyses of RR of any grade adverse events for ICI+RT in solid tumors. ICI: Immune checkpoint inhibitors; RR: relative risk; RT: radiotherapy.

**Figure 9:**
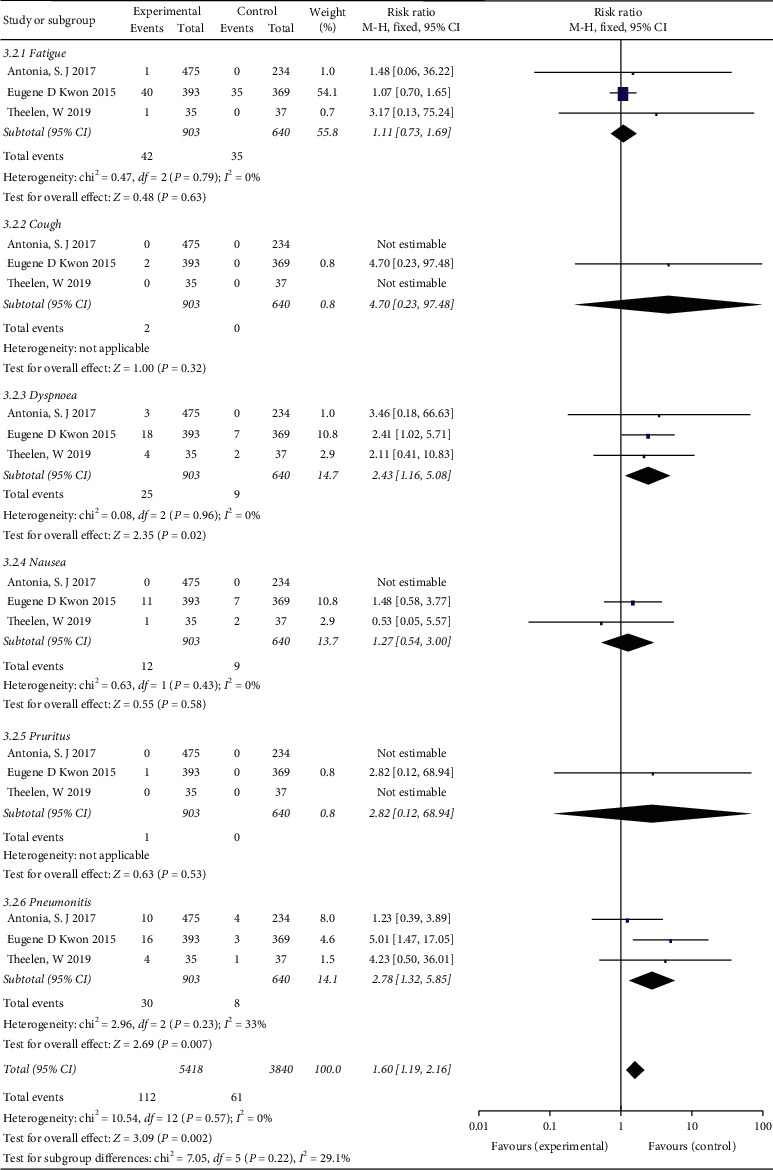
Forest plot for the subgroup analyses of RR of grade 3–5 adverse events for ICI+RT in solid tumors. ICI: Immune checkpoint inhibitors; RR: relative risk; RT: radiotherapy.

The meta-analysis pooled results of any grade AEs are presented in Figure 8. The heterogeneity test indicates that a fixed-effects model can be selected. There was minimal statistical heterogeneity in pruritus (*I*^2^ = 40%) and no heterogeneity in fatigue, cough, dyspnea, nausea, and pneumonitis (*I*^2^ = 15%, *I*^2^ = 18%, *I*^2^ = 0, *I*^2^ = 20%, *I*^2^ = 0, respectively). The results indicated that ICI plus RT led to a higher risk of fatigue (RR = 1.19; 95% CI 1.01–1.41, *P*=0.04), cough (RR = 1.55; 95% CI 1.06–2.27, *P*=0.02), pruritus (RR = 3.65; 95% CI 2.52–5.28, *P* < 0.00001), and pneumonitis (RR = 2.66; 95% CI 1.64–4.31, *P* < 0.00001) than RT with placebo/ICI alone. Differences were not statistically significant with respect to dyspnea (RR = 1.37; 95% CI 0.98–1.90, *P*=0.06) and nausea (RR = 1.05; 95% CI 0.86–1.29, *P*=0.60) between the two groups. The results of grade 3–5 AEs are described in Figure 9. More dyspnea (RR = 2.43; 95% CI 1.16–5.08, *P*=0.02) and pneumonitis (RR = 2.78; 95% CI 1.32–5.85, *P*=0.007) might occur in patients treated with ICI combined with RT compared with patients treated with RT with placebo/ICI alone, but the rates of fatigue, cough, nausea, and pruritus were not significantly different between the two groups.

## 4. Discussion

There are strong preclinical and clinical reasons for combining ICI with RT in the treatment of tumors. The aim of treatment is to achieve local and systemic disease control, which may lead to improvements in PFS and OS. However, data on the safety and efficacy of this strategy are still limited. We have conducted a systematic review of studies to explore the efficacy and safety of immunotherapy combined with RT in the treatment of solid tumors. To the best of our knowledge, this is the first systematic review and meta-analysis explicitly assessing this topic. However, the publications in this systematic review mainly report data from patients with lung cancer and prostate cancer, so these results cannot be directly translated to patients with other primary tumors.

The overarching message of our analysis is the finding that the combination of ICI with RT is effective. This is consistent with the conclusions from several studies that have examined the combination of ICI and RT. The phase III Pacific trial confirmed that the addition of durvalumab to chemoradiotherapy significantly improves both OS and PFS in lung cancer patients. Therefore, the standard treatment for stage III NSCLC now includes consolidative durvalumab [9, 12]. In a secondary analysis of the KEYNOTE-001 phase trial, patients were divided into subgroups comparing patients who had previously received RT and those who had not received RT. PFS and OS with pembrolizumab were significantly longer in patients who had previously received RT than in patients who had not previously received RT [13]. A phase I study conducted by Kelly et al. explored the efficacy and safety of atezolizumab plus stereotactic ablative radiation therapy (SBRT) for medically inoperable patients with early-stage NSCLC. The preliminary results showed that the combined treatment was a feasible option without obvious additional toxicity [14]. Moreover, the results of phase II prospective trial in patients with metastatic NSCLC reported that the combination of SBRT with concurrent pembrolizumab led to increased PFS, with a systemic response rate of 9.52% and a DCR of 57.14% [15]. At present, the results of many clinical studies, such as NCT02562625 and NCT02730130, have not been fully published [16]. The results of our meta-analysis may provide guidance for the current clinical work.

In addition, our meta-analysis of elderly subjects revealed that the risk of recurrence and metastasis was reduced by 51% upon the addition of RT to ICI in NSCLC patients aged under 65 years. These results are similar to those of previous studies of ICI [17, 18]. A previous meta-analysis explored the differential efficacy of ICI in older patients compared to young adults. In that analysis, the patients were divided into younger and older groups using a cut-off age of 65 years. PFS was not significantly different between ICI and controls for the older group (HR = 0.77; 95% CI 0.58–1.01, *P*=0.06) [17]. The potential correlation between old age and primary drug resistance in immunotherapy may be related to immunosenescence, which is a phenomenon of decreased immune function as a result of age-associated alterations to the immune system [19, 20]. The majority of cancer morbidity and mortality occur in ≥65 year-olds, but older patients were “strikingly underrepresented” in a set of RCTs [21, 22]. Most elderly patients who are excluded from RCTs have comorbid conditions, reduced performance status, organ dysfunction, etc. [23]. Therefore, combination therapy should be used cautiously in geriatric patients.

In assessing the benefits of treatment, it is important to consider the possible side effects of immunotherapy and RT. In our meta-analysis, the incidence of fatigue, cough, pruritus, and pneumonitis of any cause and grade upon combined treatment was higher than with any single treatment strategy, and the incidence of grade 3–5 dyspnea and pneumonitis was 2.43 times and 2.78 times higher, respectively. Overall, AEs are generally tolerable. Given the risk of pneumonitis, this combination should continue to be evaluated in clinical trials. A retrospective study found that the incidence of pneumonitis was highest after the integration of hypofractionated body RT into ICI [24]. Barron et al. observed an increase in the risk and severity of pneumonitis in patients with previous RT and subsequent treatment with immunotherapy (OR = 6.8; 95% CI 1.6–28.5, *P*=0.009) [25]. In their prospective study of SBRT with concurrent ICI, Tian et al. reported that concurrent lung SBRT with ICI is safe, but the risk of pneumonia should be monitored more closely [26]. Zhang et al. suggested that the application of ICI before or during thoracic RT increases the incidence of radiation pneumonia [27]. Cui et al. showed that the risk of immunotherapy-related pneumonitis was associated with prior thoracic RT and combination therapy [25, 28]. Thus, clinicians should be aware of the occurrence of pneumonitis when RT and immunotherapy are combined.

The anticancer effect of RT is mainly based on the disruption of the chemical bonds within the lipid membranes and proteins and, most importantly, between the bases in DNA [29]. Apart from direct cell killing, RT has a profound effect on the tumor microenvironment and induces the abscopal effect, which may contribute to triggering an anticancer immune response [30, 31]. For example, radiation can induce immunogenic cell death (ICD) of cancer cells. ICD is a way of cell death that releases tumor antigens and causes immune responses [32]. RT upregulates tumor-associated antigens, which are expressed on the cell surface in association with MHC I antigens; therefore, RT can also induce upregulation of MHC I molecules, which is the key to T cell activation [33, 34]. Radiation may alter the profile of MHC class I related peptides to produce new antigens, making the immune system more likely to recognize these cells [3, 35]. Additionally, RT can also cause ICD, activate dendritic cells, reduce the abundance of regulatory T cells in tumors, broaden the T cell repertoire, and increase T cell trafficking [36, 37]. This radiation property is key to its synergistic effect with ICI, antibodies targeting *T* inhibitory receptors such as CTLA-4 and PD1. Radiation induces antitumor T cells, complementing the activity of ICI [38, 39]. Radiation may also activate the antitumor immune response by triggering the stimulation of the interferon gene (STING)-mediated DNA sensing pathway [40, 41]. Hou et al. demonstrated that the deficiency of noncanonical NF-*κ*B promotes radiation-induced antitumor immunity by regulating the STING-mediated type I IFN expression [42]. Furthermore, radiation regulates immune checkpoint expression. Wu et al. showed that irradiation upregulated the expression of PD-L1 in tumor cells in in vitro and in vivo experiments, and its increase was related to the irradiation dose [43]. Similarly, Dovedi et al. demonstrated that low doses of fractionated RT led to upregulation of tumor cell expression of PD-L1 in multiple syngeneic models [44]. Thus, it is highly rational to combine RT with ICI.

However, this meta-analysis has some obvious limitations. First, there were only three trials that met the eligibility criteria, so the number of included RCTs is low. This may be because many related studies are underway. Second, although our main outcome analysis of immunotherapy plus RT treatment is biologically plausible and sound, significant heterogeneity was observed between the included studies. This may be related to the different ICI and tumor types included in our study. We have minimized its influence by using the random-effects model. Our systematic review still has some strengths: we included all published RCTs exploring the efficacy and safety of ICI combined with RT for the treatment of solid tumors and found that the association of ICI and RT provides a good DCR among the subjects, and in particular, we showed that the combination of ICI with RT is significantly better than RT alone or ICI alone, with a 36% reduced risk of recurrence and metastasis and a 19% reduced risk of death, but the incidence of pneumonia increased significantly.

## 5. Conclusion

In conclusion, our meta-analysis showed that ICI with RT significantly improved OS and PFS compared with controls in patients with NSCLC and prostate cancer. A benefit with respect to PFS was observed in NSCLC patients aged <65 years treated with combined therapy. However, the incidence of dyspnea and pneumonia of grade 3 or higher also increased. Additional studies are therefore needed to confirm the possible benefits and safety of ICI in combination with RT in patients with solid tumors.

## Figures and Tables

**Table 1:** Characteristics of the 3 studies included in the meta-analysis.

Study	RCT phase	Pathology	Line	Age	Male (%)	Treatment comparison	Case	ICI	RT site	Follow-up of ICI + RT (months)	HR (95% CI) for ICI + RT	
											OS	PFS
Antonia et al. [12]	3	NSCLC	1	<65 y 387 ≥ 65 y 322	70.1	Receive chemoradiotherapy followed by durvalumab vs. placebo	473 vs. 236	Dur:10 mg/kg Q2W	Lung	14.5	0.68 (0.47–0.997)	0.51 (0.41–0.63)

Kwon et al. [10]	3	Prostate cancer	2	<70 y 449 ≥ 70 y 350	100	Receive RT followed by ipilimumab vs. placebo	399 vs. 400	Ipi:10 mg/kg Q3W	Bone	9.9	0.85 (0.72–1.00)	0.70 (0.61–0.82)

Theelen et al. [11]	2	NSCLC	1, 2	<65 y 31 ≥ 65 y 28	57.6	Pembrolizumab after radiotherapy vs. pembrolizumab alone	36 vs. 40	Pem:200 mg/kg Q3W	Lung	23.6	0.66 (0.37–1.18)	0.71 (0.42–1.18)

*Notes*: Dur: durvalumab; Ipi: Ipilimumab; Pem: pembrolizumab. HR: hazard ratio; ICI: Immune checkpoint inhibitors; NSCLC: nonsmall-cell lung cancer; ORR: overall response rate; OS: overall survival; PFS: progression-free survival; Q2W: every 2 weeks; Q3W: every 3 weeks; RCT: randomized controlled trials; RT: radiotherapy; y: years.

## Data Availability

The datasets used and/or analyzed during the present study are available from the corresponding author on reasonable request.

## References

[1] Topalian S. L., Drake C. G., Pardoll D. M. (2015). Immune checkpoint blockade: a common denominator approach to cancer therapy. *Cancer Cell*.

[2] Demaria S., Kawashima N., Yang A. M. (2005). Immune-mediated inhibition of metastases after treatment with local radiation and CTLA-4 blockade in a mouse model of breast cancer. *Clinical Cancer Research: An Official Journal of the American Association for Cancer Research*.

[3] Reits E. A., Hodge J. W., Herberts C. A. (2006). Radiation modulates the peptide repertoire, enhances MHC class I expression, and induces successful antitumor immunotherapy. *Journal of Experimental Medicine*.

[4] Dewan M. Z., Galloway A. E., Kawashima N. (2009). Fractionated but not single-dose radiotherapy induces an immune-mediated abscopal effect when combined with anti-CTLA-4 antibody. *Clinical Cancer Research*.

[5] Postow M. A., Callahan M. K., Barker C. A. (2012). Immunologic correlates of the abscopal effect in a patient with melanoma. *New England Journal of Medicine*.

[6] Shimizu J., Yamazaki S., Sakaguchi S. (1999). Induction of tumor immunity by removing CD25+CD4+ T cells: a common basis between tumor immunity and autoimmunity. *Journal of Immunology*.

[7] Hiniker S. M., Chen D. S., Reddy S. (2012). A systemic complete response of metastatic melanoma to local radiation and immunotherapy. *Translational Oncology*.

[8] Moher D., Liberati A., Tetzlaff J., Altman D. G. (2009). Reprint-preferred reporting items for systematic reviews and meta-analyses: the prisma statement. *Physical Therapy*.

[9] Antonia S. J., Villegas A., Daniel D. (2017). Durvalumab after chemoradiotherapy in stage III non-small-cell lung cancer. *New England Journal of Medicine*.

[10] Kwon E. D., Drake C. G., Scher H. I. (2014). Ipilimumab versus placebo after radiotherapy in patients with metastatic castration-resistant prostate cancer that had progressed after docetaxel chemotherapy (CA184-043): a multicentre, randomised, double-blind, phase 3 trial. *The Lancet Oncology*.

[11] Theelen W., Peulen H., Lalezari F. (2019). Effect of pembrolizumab after stereotactic body radiotherapy vs pembrolizumab alone on tumor response in patients with advanced non-small cell lung cancer: results of the PEMBRO-RT phase 2 randomized clinical trial. *Jama Oncology*.

[12] Antonia S. J., Villegas A., Daniel D. (2018). Overall survival with durvalumab after chemoradiotherapy in stage III NSCLC. *New England Journal of Medicine*.

[13] Shaverdian N., Lisberg A. E., Bornazyan K. (2017). Previous radiotherapy and the clinical activity and toxicity of pembrolizumab in the treatment of non-small-cell lung cancer: a secondary analysis of the KEYNOTE-001 phase 1 trial. *The Lancet Oncology*.

[14] Kelly K., Daly M. E., Mirhadi A. (2020). Atezolizumab plus stereotactic ablative therapy for medically inoperable patients with early-stage non-small cell lung cancer. *Journal of Clinical Oncology*.

[15] Campbell A. M., Cai W. L., Burkhardt D. (2019). Final results of a phase II prospective trial evaluating the combination of stereotactic body radiotherapy (SBRT) with concurrent pembrolizumab in patients with metastatic non-small cell lung cancer (NSCLC). *International Journal of Radiation Oncology Biology Physics*.

[16] Shevtsov M., Sato H., Multhoff G., Shibata A. (2019). Novel approaches to improve the efficacy of immuno-radiotherapy. *Frontiers in Oncology*.

[17] Nishijima T. F., Muss H. B., Shachar S. S., Moschos S. J. (2016). Comparison of efficacy of immune checkpoint inhibitors (ICIs) between younger and older patients: a systematic review and meta-analysis. *Cancer Treatment Reviews*.

[18] Landre T., Taleb C., Nicolas P., Guetz G. D. (2016). Is there a clinical benefit of anti-PD-1 in patients older than 75 years with previously treated solid tumour?. *Journal of Clinical Oncology*.

[19] Pawelec G., Derhovanessian E., Larbi A. (2010). Immunosenescence and cancer. *Critical Reviews in Oncology/Hematology*.

[20] Ferrara R., Mezquita L., Auclin E., Chaput N., Besse B. (2017). Immunosenescence and immunecheckpoint inhibitors in non-small cell lung cancer patients: does age really matter?. *Cancer Treatment Reviews*.

[21] Murthy V. H., Krumholz H. M., Gross C. P. (2004). Participation in cancer clinical trials. *JAMA*.

[22] Ludmir E. B., Mainwaring W., Lin T. A. (2019). Factors associated with age disparities among cancer clinical trial participants. *JAMA Oncology*.

[23] Rocque G. B., Williams G. R. (2019). Bridging the data-free zone: decision making for older adults with cancer. *Journal of Clinical Oncology*.

[24] Mohamad O., Diaz D. L. A., Schroeder S. (2018). Safety and efficacy of concurrent immune checkpoint inhibitors and hypofractionated body radiotherapy. *OncoImmunology*.

[25] Barron F., Ramirez-Tirado L., Macedo-Pérez O. (2017). P3.14-012 risk of developing pneumonitis increases in patients receiving immunotherapy with a history of lung irradiation. *Journal of Thoracic Oncology*.

[26] Tian S., Switchenko J. M., Buchwald Z. S. (2020). Lung stereotactic body radiation therapy and concurrent immunotherapy: a multicenter safety and toxicity analysis. *International Journal of Radiation Oncology Biology Physics*.

[27] Zhang N., Zhu X., Kong C. (2020). 1907P Application of anti-PD1 drugs before or during thoracic radiotherapy increases the incidence of radiation pneumonia compared to the application after radiotherapy. *Annals of Oncology*.

[28] Cui P., Liu Z., Wang G. (2018). Risk factors for pneumonitis in patients treated with anti-programmed death-1 therapy: a case-control study. *Cancer Medicine*.

[29] Selzer E., Hebar A. (2012). Basic principles of molecular effects of irradiation. *Wien Med Wochenschr*.

[30] De Ruysscher D., Reynders K., Van Limbergen E., Lambrecht M. (2017). Radiotherapy in combination with immune checkpoint inhibitors. *Current Opinion in Oncology*.

[31] Wang Y., Liu Z. G., Yuan H. (2019). The reciprocity between radiotherapy and cancer immunotherapy. *Clinical Cancer Research*.

[32] Kroemer G., Galluzzi L., Kepp O., Zitvogel L. (2013). Immunogenic cell death in cancer therapy. *Annual Review of Immunology*.

[33] Kwilas A. R., Donahue R. N., Bernstein M. B., Hodge J. W. (2012). In the field: exploiting the untapped potential of immunogenic modulation by radiation in combination with immunotherapy for the treatment of cancer. *Frontiers in Oncology*.

[34] Zhang B., Bowerman N. A., Salama J. K. (2007). Induced sensitization of tumor stroma leads to eradication of established cancer by T cells. *Journal of Experimental Medicine*.

[35] Dudek A. M., Garg A. D., Krysko D. V., De Ruysscher D., Agostinis P. (2013). Inducers of immunogenic cancer cell death. *Cytokine & Growth Factor Reviews*.

[36] Meng X., Feng R., Yang L., Xing L., Yu J. (2019). The role of radiation oncology in immuno‐oncology. *The Oncologist*.

[37] Longo V., Brunetti O., Azzariti A. (2019). Strategies to improve cancer immune checkpoint inhibitors efficacy, other than abscopal effect: a systematic review. *Cancers (Basel)*.

[38] Pilones K. A., Vanpouille-Box C., Demaria S. (2015). Combination of radiotherapy and immune checkpoint inhibitors. *Seminars in Radiation Oncology*.

[39] Sindoni A., Minutoli F., Ascenti G., Pergolizzi S. (2017). Combination of immune checkpoint inhibitors and radiotherapy: review of the literature. *Critical Reviews in Oncology/Hematology*.

[40] Storozynsky Q., Hitt M. M. (2020). The impact of radiation-induced DNA damage on cGAS-STING-mediated immune responses to cancer. *International Journal of Molecular Sciences*.

[41] Reisländer T., Groelly F. J., Tarsounas M. (2020). DNA damage and cancer immunotherapy: a STING in the tale. *Molecular Cells*.

[42] Hou Y., Liang H., Rao E. (2018). Non-canonical NF-*κ*B antagonizes STING sensor-mediated DNA sensing in radiotherapy. *Immunity*.

[43] Wu C. T., Chen W. C., Chang Y. H., Lin W. Y., Chen M. F. (2016). The role of PD-L1 in the radiation response and clinical outcome for bladder cancer. *Scientific Reports*.

[44] Dovedi S. J., Adlard A. L., Lipowska-Bhalla G. (2014). Acquired resistance to fractionated radiotherapy can be overcome by concurrent PD-L1 blockade. *Cancer Research*.

